# In an escape room with my patient - a hierarchical framework for therapeutic alliance in psychosis

**DOI:** 10.1192/j.eurpsy.2023.2280

**Published:** 2023-07-19

**Authors:** M. Carneiro

**Affiliations:** Psychiatry, Centro Hospitalar e Universitário de Coimbra, Coimbra, Portugal

## Abstract

**Introduction:**

Therapeutic Alliance (TA) is a fundamental aspect of clinical practice, in particular with psychotic patients. In this population, TA has been associated with better clinical outcomes, such as symptomatic and functional impairment reduction and greater life quality. Several aspects have been suggested to negatively interfere with this patient-therapist relationship, including different beliefs about mental health/disease, the role of mental health systems or mental disease-associated stigma. Showing empathy, stimulating metacognition, searching for shared meaning and working as a team seem to be an adequate way to surpass these obstacles - just like we do when we enter an escape room.

**Objectives:**

To understand the importance of TA in the successful clinical management of psychotic patients; and to propose an original hierarchical framework of TA with these patients.

**Methods:**

Review of the scientific literature on the subject and proposal of an original framework of TA with psychotic patients doing an analogy with escape room dynamics.

**Results:**

In an escape room, a team works together to solve a series of mysteries, each leading to another, thus accomplishing one final goal. To do such, every element must agree on the solution to proceed; and there is no access to one level without successfully completing the former.

In a clinical environment with a psychotic patient, such rules may also apply. Although the starting level in each patient-therapist relationship may vary, it is here proposed that proceeding to the next level without at least partial agreement in the previous one may be inefficacious or even counterproductive for the intentions of the therapist.

So, shall we begin?

Level 0 - Who am I? Prior to the encounter with the patient, it is important that the therapist knows himself reasonably well, specially regarding his beliefs, shortcomings and biases in his clinical practice.

Level 1 - What is this? The teamwork starts here. Both therapist and patient must discover why this encounter is happening, what does it mean to be in this medical facility or to be mentally ill, in general.

Level 2 - What is wrong? Trying to understand what is detrimental in the patient’s mental health.

Level 3 - What to do? Tailoring a therapeutic intervention to the impairments of the patient.

Level 4 - How to do it? Finding a way to suit the therapeutic intervention to patient’s personal preferences and availability.

Level 5 - The way out. Helping the patient escape this room and fulfill his maximum potential in all of the other “rooms” of his life.

**Conclusions:**

TA is indispensable to therapeutic success when dealing with psychotic patients.

There is a need for shared meaning and understanding in multiple aspects of the therapist-patient relationship; an approach that only focus some of these aspects while ignoring others may be ineffective or even detrimental to clinical outcomes.

**Disclosure of Interest:**

None Declared

